# Low Systolic Blood Pressure and Mortality in Elderly Patients After Acute Myocardial Infarction

**DOI:** 10.1161/JAHA.119.013030

**Published:** 2020-02-26

**Authors:** Basile Mouhat, Alain Putot, Olivier Hanon, Jean Christophe Eicher, Frédéric Chagué, Jean‐Claude Beer, Maud Maza, Marianne Zeller, Yves Cottin, Observatoire des Infarctus de Côte d'Or Survey

**Affiliations:** ^1^ Service de Cardiologie CHU Dijon Dijon France; ^2^ Médecine Interne Gériatrie Pôle Personnes Âgées Centre Hospitalier Universitaire de Dijon Dijon France; ^3^ Service de Gériatrie Assistance Publique – Hôpitaux de Paris Hôpital Broca Paris France; ^4^ EA 4468 Université Paris Descartes Sorbonne Paris Cité, Paris France; ^5^ Laboratoire Physiopathologie et Epidémiologie Cérébro‐Cardiovasculaires EA 7460 Université de Bourgogne Franche‐Comté Dijon France

**Keywords:** acute myocardial infarction, blood pressure, cardiovascular mortality, elderly patients, Myocardial Infarction, Epidemiology

## Abstract

**Background:**

Optimal blood pressure in elderly patients after acute myocardial infarction is still a matter of debate. In a prospective observational study, we aimed to identify optimal systolic blood pressure during the 48 first hours after admission for acute myocardial infarction and its prognostic value for cardiovascular mortality.

**Methods and Results:**

From the Observatoire des Infarctus de Côte d'Or survey, all consecutive patients aged >75 years admitted for an acute myocardial infarction in a coronary care unit from 2012 to 2015 and discharged alive were included (n=814). Exclusion criteria were in‐hospital death, cardiogenic shock, and end‐stage renal disease. Average systolic blood pressure (aSBP) values over the first 48 hours after admission were recorded, and the population was dichotomized into 2 groups: low aSBP group (<125 mm Hg) and control group (aSBP ≥125 mm Hg). When compared with patients without cardiovascular death at 1‐year follow‐up, patients who died from a cardiovascular cause had higher rate of cardiovascular risks factors, including age, diabetes mellitus, comorbidities, and cardiovascular history. They had higher rates of low body mass index (<21 kg/m^2^) and more elevated Global Registry of Acute Coronary Events risk score. Patients with aSBP <125 mm Hg had a 2‐fold risk of 1‐year cardiovascular death (47 [12.0%] versus 28 [6.6%]; *P*=0.008). By multivariable logistic regression analysis, low aSBP (odds ratio [95% CI], 1.91 [1.07–3.41]) remained a strong and independent predictor of 1‐year cardiovascular mortality.

**Conclusions:**

In our large population‐based study in elderly patients with acute myocardial infarction, low aSBP was an independent and powerful predictor of 1‐year cardiovascular mortality. Early aSBP measurement could help to improve risk stratification. Moreover, our results may suggest an optimal blood pressure target in elderly patients.

**Clinical Perspective**

**What Is New?**

- This work identifies average systolic blood pressure, a simple hemodynamic parameter to be collected by the clinician, as a new prognostic marker for cardiovascular mortality in elderly patients with acute myocardial infarction (MI).
- In patients aged >75 years, our data showed that low average systolic blood pressure during the first 48 hours after MI is associated with poor prognosis at 1 year.
- The average systolic blood pressure provides additional information on the prognosis, beyond the GRACE (Global Registry of Acute Coronary Events) score.

**What Are the Clinical Implications?**

- Our study suggests that after acute MI, closer follow‐up of these at‐risk patients is needed.
- Introduction and titration of hypotensive therapies at the short‐term phase of management should be considered with caution in elderly patients after acute MI.
- Our findings suggest the benefit of maintaining average systolic blood pressure >125 mm Hg within the first 48 hours after MI.

Population aging is accelerating rapidly worldwide. By 2050 in the United States, the number of older people is projected to increase by 135% and the population aged ≥85 years by 350%.^1^

Acute myocardial infarction (AMI) is a frequent cause of hospital admission in patients aged >75 years. They represented ≈40% of hospitalizations for AMI in 2013 in France.^2^ In AMI, advanced age is a powerful predictor of both early and long‐term mortality. In‐hospital death rates were 17% in patients aged >85 years in the EuroHeart survey^3^ versus <1% in younger patients, and 1‐year all‐cause mortality was ≈20% for those aged >75 years in the FAST‐MI (French Registry of Acute ST‐Elevation or Non–ST‐elevation Myocardial Infarction) registry.^4^

Lowering blood pressure (BP) in primary prevention in elderly patients is an important part of the management of cardiovascular risk factors and has relative safety and efficacy, recently confirmed by randomized clinical trials.^5^, ^6^ The optimal target for BP in elderly patients remains unclear; and a new systolic BP (SBP) target at <120 mm Hg was recently supported by the SPRINT (Systolic Blood Pressure Intervention Trial) for those aged >75 years, showing a significant cardiovascular benefit and further leading to lower BP target up to 130/80 mm Hg in the current US recommendations.^6^

Low SBP at AMI admission, after hospitalization, or in stable coronary artery disease is usually associated with a poor prognosis.^7^, ^8^ However, no study has specifically been performed to identify optimal SBP target in the short‐term phase of AMI in elderly patients. The aim of the present work was to analyze SBP at the early phase of AMI as a prognostic marker in elderly patients.

## Materials and Methods

The data that support the findings of this study are available from the corresponding author (Marianne.zeller@u-bourgogne.fr) on reasonable request.

### Study Design

From the Observatoire des Infarctus de Côte d'Or survey database, all consecutive patients aged >75 years with ST‐segment–elevation MI (STEMI) or non‐ST‐segment‐elevation MI (NSTEMI) admitted in the coronary care unit (CCU) of Dijon, France, between February 1, 2012, and January 31, 2015, were included. Methods and study population of the French regional Observatoire des Infarctus de Côte d'Or survey have been described elsewhere.^9^ Briefly, Observatoire des Infarctus de Côte d'Or is an ongoing survey that prospectively collects data from patients hospitalized for AMI in all public centers or privately funded hospitals of one eastern region of France. AMI, including STEMI or NSTEMI, was diagnosed according to the European Society of Cardiology and the American College of Cardiology criteria.^10^ Exclusion criteria were: in‐hospital death; cardiogenic shock, as currently defined,^11^ or intra‐aortic balloon pump counterpulsation within the first 48 hours; cardiac arrest; and end‐stage renal disease (creatinine clearance <15 mL/min per 1.73 m^2^ or need for hemodialysis) because of potential hemodynamic instability. These criteria aimed to exclude conditions of major BP fluctuations and poor prognostic disease. A small subset of patients’ files who died in the hospital (n=28) were analyzed and confirmed BP instability. This strengthens the relevance of exclusion criteria and that no useful information could be added from these excluded patients. The present study complied with the Declaration of Helsinki and was approved by the Ethics Committee of Dijon University Hospital. Each patient gave written consent before participation.

### Data Collection

Demographic data, cardiovascular risk factors, and history were collected, as were on‐admission ECG, clinical data, and biological data. The GRACE (Global Registry of Acute Coronary Events) risk score at discharge was also calculated.^12^ The Charlson Comorbidity Index (CCI) was calculated for each patient to classify comorbid conditions. Blood samples were taken on admission to measure NT‐proBNP (N‐terminal pro‐B‐type natriuretic peptide), serum creatinine, and CRP (C‐reactive protein). Troponin Ic peak was provided from 3 samplings every 8 hours for <24 hours. Estimated glomerular filtration rate was calculated using the Chronic Kidney Disease–Epidemiology Collaboration formula. Left ventricular ejection fraction (LVEF) was measured by echocardiography at 2±2 days. Long‐ and short‐term medications (<48 hours after admission), antihypertensive treatments, and the rate of revascularization (coronary artery bypass grafting [CABG] or primary percutaneous coronary intervention [PCI]) were also reported.

### Hemodynamic Measurement and Average SBP

Heart rate and BP were routinely measured by a semiautomatic sphygmomanometer (Philips System Med) at least 3 times a day (6:00 am, 12:00 am, and 6:00 pm). Average SBP (aSBP) values were calculated from ≥3 SBP values within the first 48 hours of the hospitalization in CCU. At least 3 measurements were collected for each patient, with a mean of 5.4±1.0 measurements. aSBP was 126.6±16.3 mm Hg, ranging from 84.8 to 183.8 mm Hg. Average diastolic BP, mean blood pressure, and pulse pressure were also calculated.

### Study Outcomes

One‐year events were recorded by mailing or telephone interview to the patients, family, or general practitioner, including cardiovascular death, CABG, unstable angina, angina pectoris, stroke, recurrent MI, heart failure, or unscheduled PCI. Cardiovascular mortality was defined by death caused by sudden death, heart failure, recurrent AMI, fatal arrhythmia (ventricular tachycardia or ventricular fibrillation), or stroke.

One‐year cardiovascular mortality was the primary outcome. The secondary outcome was a composite of death attributable to any cause, hospitalization for heart failure, unscheduled PCI or CABG, unstable angina, angina pectoris, stroke, or recurrent MI, at 1 year.

### Statistical Analysis

Qualitative variables were expressed as number (percentage), and continuous variables were expressed as median (interquartile range). A Kolmogorov‐Smirnov test was performed to analyze the normality of continuous variables. A Mann‐Whitney or Student *t* test was used to compare continuous data, and the χ^2^ test or Fisher test was used for dichotomous data, as appropriate. Pearson or Spearman tests were used to assess the correlation for continuous variables. Significance threshold was set at 5%.

To identify the more relevant BP parameter, we first compared the predictive values of aSBP, mean arterial pressure, and diastolic BP as estimates for 1‐year cardiovascular mortality, using the concordance index (C statistic). The C statistic with aSBP was higher than with diastolic BP (0.55 versus 0.53), suggesting a higher predictive value, and the C statistics for average mean arterial pressure and aSBP were similar. Because mean arterial pressure is more complex than aSBP to collect at bedside, we choose to use aSBP.

Receiver operating curve analysis was then set to identify the optimal cutoff, using the Youden index method. Area under the curve value was at 0.55 (95% CI, 0.48–0.62), with an optimal aSBP cutoff value at 125 mm Hg. This cutoff value was in agreement with the current literature^8^, ^9^ and was thus kept throughout the analysis.

Patients were thus dichotomized into 2 groups, according to the aSBP value (≥125 or <125 mm Hg). A multivariable logistic regression model was built to estimate 1‐year cardiovascular death, including confounding factors identified from the literature and from univariate analysis (body mass index <21 kg/m^2^, diabetes mellitus, prior stroke and/or coronary artery disease, STEMI with anterior location, LVEF <40%, GRACE risk score at discharge, and CCI class). Low SBP (ie, aSBP <125 mm Hg) as a variable was introduced as part of the stepwise procedure. Inclusion threshold was set at 5%, and variable selection was stepwise and *P* value based. SPSS, version 12.0.1 (IBM Inc), was used for all of the statistical tests.

## Results

**Figure 1:**
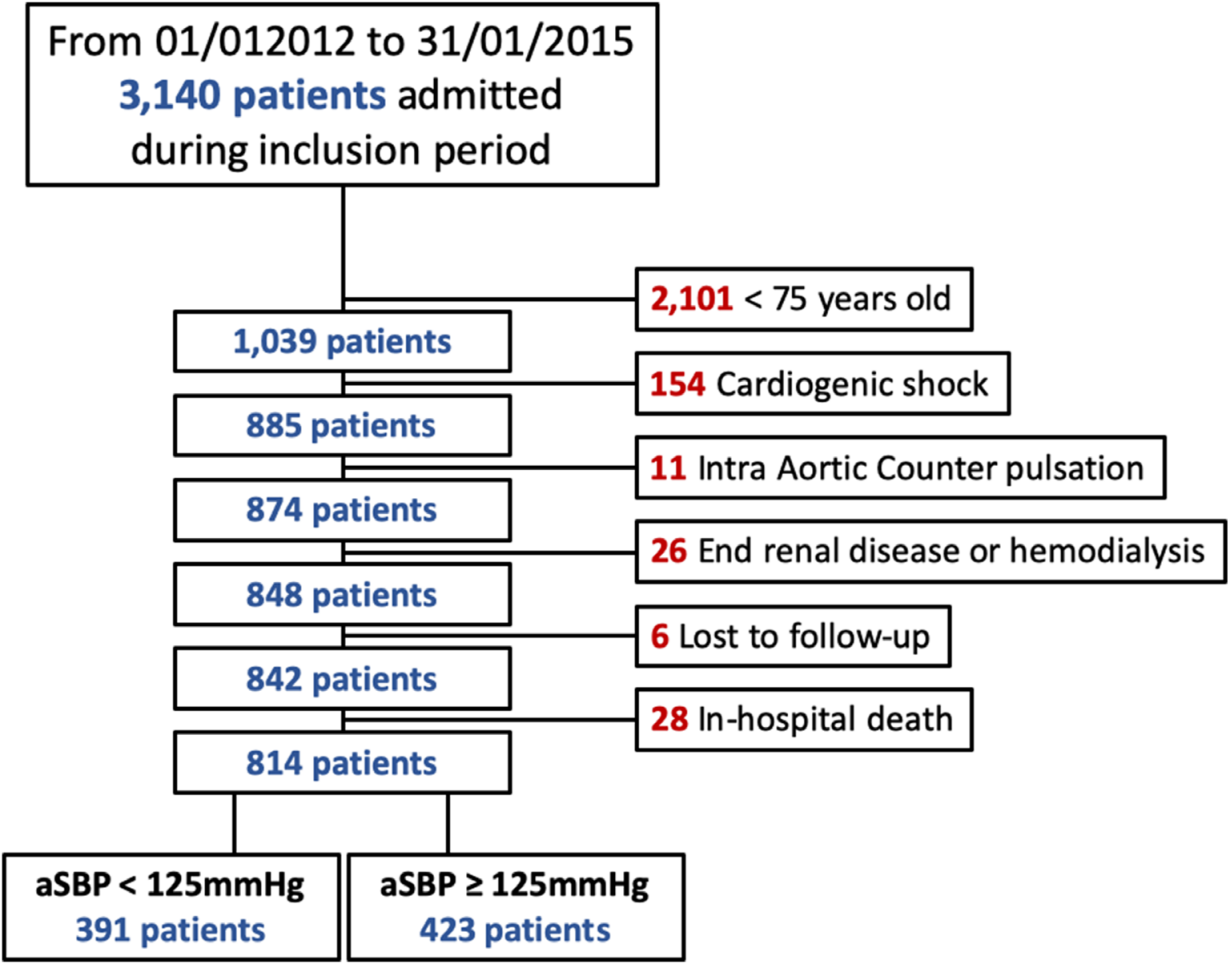
Study flowchart. aSBP indicates average systolic blood pressure.

During the inclusion period, 3140 patients were admitted in the Dijon CCU for an AMI, including 1039 patients aged >75 years, of whom 814 were included in the present analysis, after exclusion criteria or patients who died in the hospital. Six patients were lost to follow‐up. Among the remaining 814 patients, 391 (48%) had aSBP <125 mm Hg, as presented in Figure 1.

### Baseline Characteristics

Baseline characteristics, stratified by outcome status (ie, with or without cardiovascular death at 1 year), are reported in Table 1. Median age was 82 years, and 51% were women. When compared with patients without cardiovascular death at 1 year, patients who died because of a cardiovascular cause had higher rate of cardiovascular risks factors, including age and diabetes mellitus. They had higher rates of low body mass index (<21 kg/m^2^); comorbidities, as assessed by CCI class; stroke and/or coronary artery disease; prior heart failure; chronic renal disease; and aortic stenosis. Admission parameters showed elevated heart rate, higher rate of in‐hospital heart failure, more frequent altered LVEF (ie, <40%), and higher GRACE risk score at discharge in patients with cardiovascular death. Biological admission parameters showed more frequent altered renal function, higher NT‐proBNP values, and more elevated CRP in the group of patients dead because of cardiovascular cause at 1 year. Table 1 also shows that patients without cardiovascular death at 1 year less often had β blockers, diuretics, aspirin, and statins as long‐term medications.

**Table 1:** Baseline Patients’ Characteristics According to Outcomes at 1‐Year Follow‐Up

Characteristics	No Cardiovascular Death (n=739)	Cardiovascular Death (n=75)	*P* Value
Risk factors			
Age, y	82 (79–86)	85 (81–89)	0.001
Women	380 (51)	34 (45)	0.394
BMI <21 kg/m^2^	84 (11)	17 (23)	0.008
Hypertension	575 (78)	65 (87)	0.052
Hypercholesterolemia	367 (50)	42 (56)	0.244
Family history of CAD	167 (23)	11 (15)	0.125
Diabetes mellitus	180 (24)	33 (44)	<0.001
Smoking	38 (5)	5 (7)	0.554
Cardiovascular history			
Stroke	89 (12)	16 (21)	0.019
CAD	168 (23)	23 (31)	0.106
Prior stroke and/or CAD	235 (32)	36 (48)	0.003
Heart failure	66 (9)	15 (20)	0.002
Chronic renal failure	51 (7)	11 (15)	0.014
Aortic stenosis	77 (10)	15 (20)	0.011
Long‐term medications			
β Blockers	296 (40)	39 (52)	0.035
ACE inhibitors or ARBs	378 (51)	46 (61)	0.071
Diuretics	150 (20)	27 (36)	0.001
Potassium‐sparing diuretics	25 (3)	4 (5)	0.371
Thiazides	130 (18)	17 (23)	0.251
Aspirin	242 (33)	33 (44)	0.040
P2Y12 receptor antagonists	104 (14)	15 (20)	0.150
Statin	235 (32)	32 (43)	0.046
Biological data			
CKD‐eGFR <60 mL/min per 1.73 m²	365 (49)	49 (65)	0.007
NT‐proBNP, pg/mL	1790 (528–5234)	5510 (3050–14 705)	<0.001
Troponin I peak, μg/L	6.8 (1.7–32.0)	7.3 (2.2–49)	0.231
CRP >3 mg/L	486 (66)	65 (87)	<0.001
Clinical data			
CCI class			
CCI‐0 (0)	248 (34)	10 (13)	0.001
CCI‐1 (1–2)	288 (39)	31 (41)	
CCI‐2 (3–4)	116 (16)	17 (23)	
CCI‐3 (>5)	87 (12)	16 (21)	
HR at admission, beats/min	79 (69–90)	88 (70–103)	0.006
SBP, mm Hg			
At admission	143 (123–160)	130 (115–150)	0.002
aSBP <125 mm Hg	344 (47)	46 (61)	0.010
DBP, mm Hg			
At admission	76 (65–74)	70 (63–84)	0.269
Heart failure at admission	71 (10)	14 (19)	0.013
STEMI	281 (38)	27 (36)	0.795
LVEF <40%	112 (15)	30 (40)	<0.001
Length of ICU stay, d	4 (3–5)	4 (3–6)	0.278
GRACE risk score at discharge	137 (126–150)	153 (139–173)	<0.001
Time to admission, min	209 (105–600)	270 (89–1005)	0.507
Heart failure in hospital	105 (14)	25 (33)	<0.001

Data are given as median (interquartile range) or number (percentage). ACE indicates angiotensin‐converting enzyme; ARB, angiotensin receptor blocker; aSBP, average SBP; BMI, body mass index; CAD, coronary artery disease (including unstable angina, myocardial infarction, coronary artery bypass grafting, and percutaneous coronary intervention); CCI, Charlson Comorbidity Index; CKD, chronic kidney disease; CRP, C‐reactive protein; DBP, diastolic blood pressure; eGFR, estimated glomerular filtration rate; GRACE, Global Registry of Acute Coronary Events; HR, heart rate; ICU, intensive care unit; LVEF, left ventricular ejection fraction; NT‐proBNP, N‐terminal pro‐B‐type natriuretic peptide; SBP, systolic blood pressure; STEMI, ST‐segment–elevation myocardial infarction.

### Short‐Term Therapies

The group without cardiovascular death at 1 year underwent more often coronary angiography (92% versus 81%; *P*=0.001) and revascularization (Table 2). Within the first 48 hours, they had fewer diuretics (41% versus 67%; *P*<0.001) and more angiotensin‐converting enzyme or angiotensin receptor blockers (76% versus 62%; *P*=0.010). Also, they had fewer diuretics (34% versus 57%; *P*<0.001) at discharge.

**Table 2:** Therapies According to Outcomes at 1‐Year Follow‐Up

Therapies	No Cardiovascular Death (n=739)	Cardiovascular Death (n=75)	*P* Value
Revascularization			
Coronary angiography	682 (92)	60 (80)	0.001
CABG	30 (4)	0 (0)	0.077
PCI	419 (57)	31 (41)	0.015
<48 h			
β Blockers	521 (71)	48 (64)	0.313
ACE inhibitors or ARBs	362 (49)	40 (53)	0.406
Diuretics	304 (41)	51 (68)	<0.001
Aspirin	714 (97)	71 (95)	0.763
P2Y12 receptor antagonists	680 (92)	65 (87)	0.216
Statins	674 (91)	63 (84)	0.087
At discharge			
β Blockers	640 (87)	58 (77)	0.053
ACE inhibitors or ARBs	560 (76)	46 (61)	0.010
Diuretics	254 (34)	42 (56)	<0.001
Aspirin	673 (91)	64 (85)	0.197
P2Y12 receptor antagonists	484 (65)	43 (57)	0.205
Statins	665 (90)	67 (89)	0.879

Data are given as number (percentage). ACE indicates angiotensin‐converting enzyme; ARB, angiotensin receptor blocker; CABG, coronary artery bypass grafting; PCI, percutaneous coronary intervention.

### Outcomes and Mortality at 1 Year

**Figure 2:**
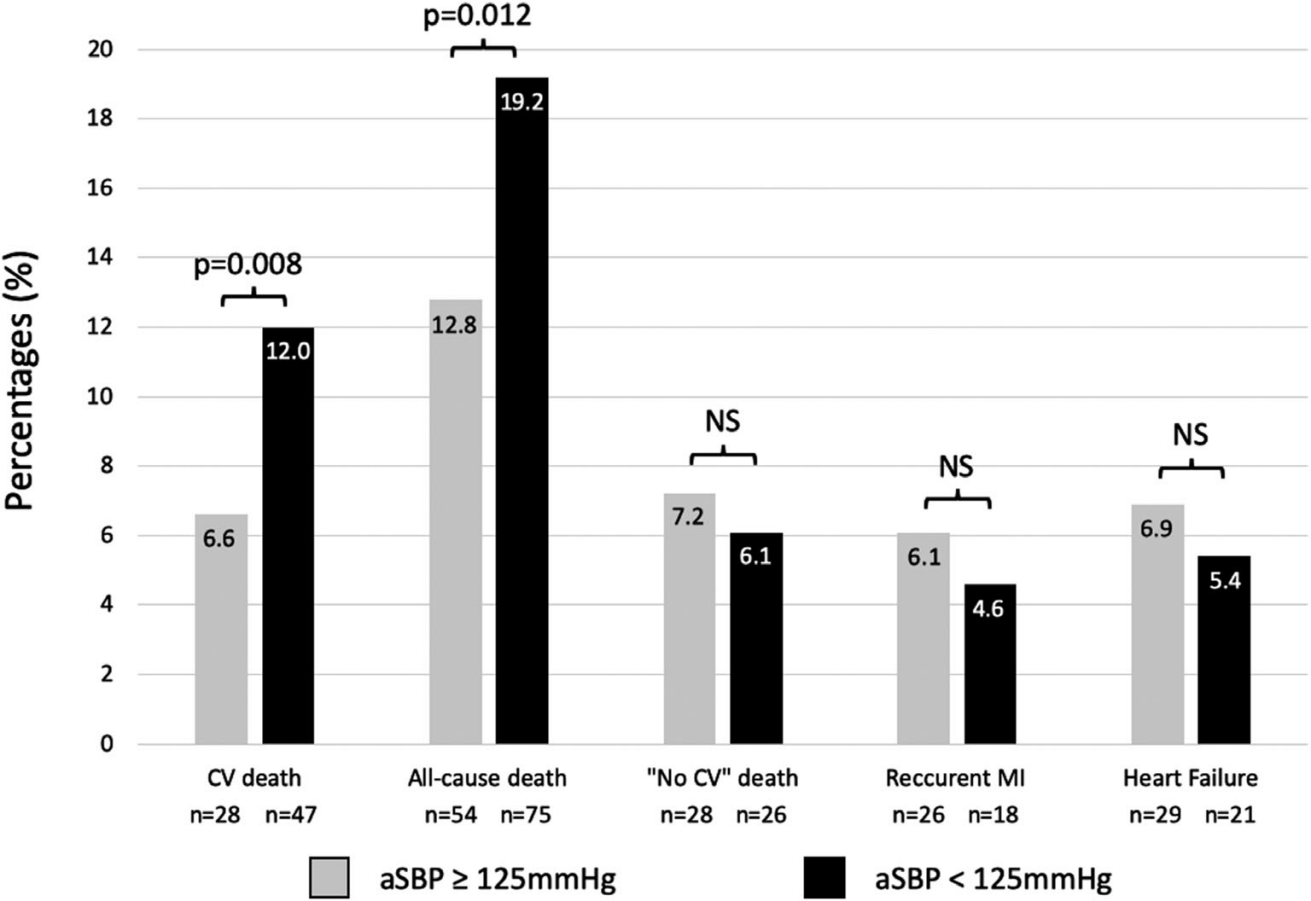
Cardiovascular outcomes at 1 year, according to average systolic blood pressure (aSBP) assessed within the first 48 hours in the coronary care unit. CV indicates cardiovascular; MI, myocardial infarction; NS, not significant.

Results are presented in Figure 2. One‐year cardiovascular death rate was almost twice higher in the aSBP <125 mm Hg group (47 [12.0%] versus 28 [6.6%]; *P*=0.008). Moreover, all‐cause death was also more frequent (75 [19.2%] versus 54 [12.8%]; *P*=0.012). Cardiovascular events, including CABG, recurrent MI, unscheduled PCI, or rehospitalization for heart failure, were similar for both groups (17 [4.3%] versus 31 [7.3%] [*P*=0.071]; 18 [4.6%] versus 26 [6.1%] [*P*=0.331]; 6 [1.5%] versus 7 [1.7%] [*P*=0.891]; and 21 [5.4%] versus 29 [6.9%] [*P*=0.378], respectively).

### Multivariate Logistic Regression Analysis

Multivariable regression analysis showed that aSBP <125 mm Hg (odds ratio [OR] [95% CI], 1.91 [1.07–3.41]; *P*=0.028) remained an independent predictor of 1‐year cardiovascular mortality, even after adjustment for covariates (ie, diabetes mellitus: OR [95% CI], 2.83 [1.47–5.43]; LVEF <40%: OR [95% CI], 2.44 [1.36–4.36]; body mass index <21 kg/m^2^: OR [95% CI], 2.24 [1.11–4.55]; and GRACE risk score at discharge: OR [95% CI], 1.04 [1.02–1.05]) (Table 3). The prognostic value of low aSBP remained significant even after exclusion of the GRACE risk score from the model and after replacement by the individual score components (ie, age, heart rate, and estimated glomerular filtration rate <60 mL/min per 1.73 m^2^).

**Table 3:** Logistic Regression Analysis for 1‐Year Cardiovascular Death Estimate

Variables	Univariable Analysis			Multivariable Analysis		
	OR	95% CI	*P* Value	OR	95% CI	*P* Value
aSBP <125 mm Hg	1.89	1.15–3.08	0.011	1.91	1.07–3.41	0.028
Age, y	1.08	1.04–1.13	0.001	···	···	···
BMI <21 kg/m^2^	2.33	1.29–4.18	0.005	2.24	1.10–4.55	0.025
Hypertension	2.06	1.00–4.26	0.049	···	···	···
Diabetes mellitus	2.50	1.53–4.07	<0.001	2.83	1.47–5.43	0.002
Prior stroke and/or CAD	1.53	1.12–2.08	0.007	1.27	0.94–1.72	0.124
Prior heart failure	2.59	1.39–4.82	0.003	···	···	···
CKD‐eGFR <60 mL/min per 1.73 m²	1.98	1.20–3.28	0.008	···	···	···
CRP >3 mg/L	3.76	1.84–7.67	<0.001	···	···	···
STEMI with anterior wall location	1.61	0.92–2.83	0.099	1.80	0.92–3.52	0.085
LVEF <40%	3.82	2.30–6.33	<0.001	2.44	1.36–4.36	0.003
HR at admission, beats/min	1.02	1.01–1.02	0.001	···	···	···
GRACE risk score at discharge	1.04	1.03–1.05	<0.001	1.04	1.02–1.05	<0.001
CCI class	1.57	1.24–1.97	<0.001	0.99	0.87–1.13	0.875

aSBP indicates average systolic blood pressure; BMI, body mass index; CAD, coronary artery disease (including unstable angina, myocardial infarction, coronary artery bypass grafting, and percutaneous coronary intervention); CCI, Charlson Comorbidity Index; CKD, chronic kidney disease; CRP, C‐reactive protein; eGFR, estimated glomerular filtration rate; GRACE, Global Registry of Acute Coronary Events; HR, heart rate; LVEF, left ventricular ejection fraction; OR, odds ratio; STEMI, ST‐segment–elevation myocardial infarction.

## Discussion

In this population‐based study in elderly patients after AMI, we showed that low aSBP within 48 hours after admission was strongly associated with an increased risk of cardiovascular death occurring after hospital discharge. This association remained significant even after adjustment for potential confounders. This preliminary study on contemporary data analyzing the relationship between early SBP level and mortality after AMI may fuel the debate about the optimal BP target in elderly patients. Indeed, after a cardiovascular event, the geriatric population is more prone to cardiovascular events related to low BP.

Our results are consistent with literature reports. Old patients received optimal treatment, including revascularization procedures. In our work, >90% of patients underwent coronary angiography, and 55% were treated by PCI (89% in STEMI and 44% in NSTEMI). This use of PCI is superior to the rate in patients aged >80 years from the randomized After Eighty Study (Invasive versus conservative strategy in patients aged 80 years or older with non–ST‐elevation myocardial infarction or unstable angina pectoris) (47%). The rate of revascularization (PCI or CABG) (59%) was even higher than in the large CRUSADE (can rapid risk stratification of unstable Angina Patients Suppress Adverse Outcomes With Early Implementation Of The ACC/AHA Guidelines) study, performed for 40% of patients aged 75 to 89 years (n=46 270).^13^ Also, the rate of drugs at discharge was high and similar to the intensive group from the After Eight Study (ie, β blockers [85%], aspirin [>90%], and statin [90%]) or even greater for angiotensin‐converting enzyme inhibitors or angiotensin receptor blockers. Second, our population has mortality consistent with the After Eight Study^14^ and the FAST‐MI registry^15^ (16% versus 26% and 20%, respectively).

The low aSBP group experienced more severe MI injury, as suggested by increased troponin peak level, more STEMI and anterior wall location, and lower LVEF. Impaired LVEF is a major predictor of mortality after AMI.^16^ Impaired LVEF and low BP are also important predictors of death in heart failure.^17^, ^18^ In our study, LVEF showed a weak but significant correlation (*r*=0.22; *P*<0.001) with aSBP. We checked the absence of multicollinearity in the logistic regression analysis model. Moreover, the interaction of LVEF <40% and aSBP <125 mm Hg, as a covariate in the final model, did not change the prognostic value of a low aSBP (OR [95% CI], 2.25 [1.11–4.54]; *P*=0.024). Altogether, these data strongly suggest that the impact of aSBP on the outcomes is at least partly independent from LVEF and brings additive information.

Another question arises as to whether the low aSBP group was overtreated. European Society of Cardiology guidelines for management of AMI^19^, ^20^ recommend treatments by β blockers within the first 24 hours in patients with LVEF <40% and/or heart failure after stabilization. Evidence is based on benefit achieved in the first week and derived from meta‐analysis.^21^ Recently, β blockers showed a 90‐day positive effect in elderly patients after AMI on death and rehospitalization, without any increase in functional decline in case of initial intact cognition or middle dementia (Mini‐Mental State Examination score >14/30).^22^ Although β blockers are essential, the optimal dose remains unclear and failed to be associated with decreased mortality.^23^ A systematic overview of trials on angiotensin‐converting enzyme inhibition started early in STEMI indicated that this therapy is safe, well tolerated, and associated with a small but significant reduction in 30‐day mortality, with most of the benefit observed within the first week.^24^ However, the recommended target value of <120 mm Hg in elderly patients without frailty after AMI remains to be confirmed. Our results strongly suggest that this threshold within the first 48 hours after AMI could be higher.

Our study has some limitations. Some data are missing, such as orthostatic hypotension, known as a major predictor of mortality in elderly patients,^25^ but also the evolution of BP after discharge, risk factor control, 1‐year cardiovascular treatments, and adherence to treatments. In addition, because this is an observational study, we cannot rule out the possibility of confounding. Stratification of comorbidity and frailty in this elderly population is a key issue of the analysis. Frailty is defined by a state of increased vulnerability, to poor resolution of homoeostasis after a stressor event, which increases the risk of adverse outcomes, including falls, delirium, and disability.^26^ We estimated the frailty through the comorbidities burden using the CCI classification. This index has a prognostic value at 30 days and 1 year after AMI.^27^ Despite prediction for 1‐year cardiovascular mortality in univariate analysis, CCI score lost significance when covariates were introduced in the multivariable model. However, this score omits nutritional status, which was estimated by body mass index <21 kg/m^2^ and appears as an independent predictor for 1‐year mortality. Confusion, functional decline, and cognition have not been assessed, although dementia is associated with lower BP and increased mortality.^28^ Anemia and bleeding are also comorbidities of bad forecast in elderly patients, but our study did not show any difference between the groups.

Nevertheless, aSBP is a simple hemodynamic parameter to monitor in CCU and could help to identify patients at risk for cardiovascular death in middle‐term, beyond traditional risk factors and LV dysfunction. However, underlying mechanisms for the association between increased risk of 1‐year cardiovascular death and low mean SBP in CCU are unclear, are probably multifactorial, and cannot be extrapolated from our observational findings. Recent study in stable coronary artery disease showed association between BP variability and cardiovascular mortality, which may be explained by direct endothelial damage and overcoming the autoregulation capacities of target organs, further leading to cardiovascular events.^29^ Alternative pathophysiological mechanisms involve altered baroreflex sensitivity and autonomic dysfunction after AMI, leading to poor adverse prognosis.^30^ The variation of BP before AMI (pre‐MI) and during AMI could even be more strongly related to the outcome. Some pre‐MI data were retrospectively collected in a subgroup of 81 patients who had BP measurement on the setting of Dijon hospital consultation (median time delay between pre‐MI and AMI, 38 days). In this subgroup, we performed a series of analyses to (1) evaluate the variations of BP parameters (including SBP, diastolic BP, mean arterial pressure, and pulse pressure) and (2) compare the prognostic value of these parameters (absolute or difference pre‐MI versus AMI). We found a decrease for most BP parameters (pre‐MI versus AMI), which were not significantly associated with 1‐year cardiovascular death. Given the weak statistical power of this subgroup analysis, we cannot exclude that the variations in BP may be more relevant to predict the outcome.

The C statistic of the GRACE risk score was higher than the aSBP (0.72 versus 0.55) for prediction of the 1‐year cardiovascular mortality. However, low aSBP had an additive and independent prognostic value for 1‐year cardiovascular mortality, without any correlation with the GRACE risk score (*r*=0.08; *P*=0.030). This simple hemodynamic parameter is efficient for risk stratification of 1‐year cardiovascular mortality within the first 48 hours after AMI in our elderly patients. Our work raises the question of the treatment strategy in the short‐term phase of MI. However, our work was not designed for this purpose but our analyses show a trend toward a positive effect of short‐term β blockers or bradycardic calcium channel blocker (<48 hours), even in the low aSBP group, without reaching the threshold of statistical significance (OR [95% CI], 0.67 [0.41–1.09]; *P*=0.108).

## Conclusions

Our findings from elderly population‐based data emphasize the strong and independent association of a low early‐addressed SBP in the intensive care unit after AMI with 1‐year cardiovascular mortality. If confirmed, these findings could help to identify high‐risk patients. Further experimental studies are needed to better understand the underlying mechanisms.

## Sources of Funding

This work was supported by the University Hospital of Dijon, the Association de Cardiologie de Bourgogne, and by grants from the Agence Régionale de Santé de Bourgogne Franche Comté and from the Conseil Régional de Bourgogne Franche Comté.

## Disclosures

Dr Cottin reports having received grants, consulting fees, or honoraria and/or delivering lectures for Servier, Novartis, Boehringer, Pfizer, MSD, and Bayer. The remaining authors have no disclosures to report.
